# A Pragmatic Approach to define “DIFFICULT TO TREAT” patients

**DOI:** 10.1192/j.eurpsy.2023.1145

**Published:** 2023-07-19

**Authors:** M. Nascimento, A. Lourenço, A. L. Falcão, G. Soares, C. Rodrigues, J. Petta, C. Oliveira

**Affiliations:** 1 Clinica 3; 2 Reabilitação/Residentes, Centro Hospitalar Psiquiátrico de Lisboa, Lisbon, Portugal

## Abstract

**Introduction:**

Multiple definitions for “difficult to treat” patients (DTP) were given throughout the years. While most authors focus on diagnoses, others focus on clinical, social and demographic factors, which should be regarded as factors of bad prognosis and elevated costs for the healthcare systems.

**Objectives:**

To identify and haracterize DTP patients admitted in acute ward, based on practical criteria.

**Methods:**

Through the hospital’s IT services, all acute inpatient episodes at Centro Hospitalar Psiquiátrico de Lisboa were collected, since 2017. Cluster analysis was performed, regarding number of previous admissions (PA) and days of admission. Descriptive and comparative statistics (with multiple comparisons) for the different clusters, regarding age, gender, diagnosis at discharge (according to ICD10), and, to the DTP, previous medical following, compliance to medication, and substance use at admission.

**Results:**

Three clusters were identified: (C1, n=5861) a larger, uncharacteristic one; (C2, n=1168) with a higher number of PA (average of 8, versus less than 2 on the others); and (C3, n=1462) with higher number of days of admissions (58 versus less than 16). Statistical significance was found regarding age (higher in C3), gender (more men in C2), nationality (C1 with more foreigners). Regarding diagnosis at discharge, statistical difference was found between the 3 groups: C1 has significantly less patients with Schizophrenia (11% versus 30% in the others), but more depressive (21% versus 6% in C2 and 12% in C3) and neurotic disorders. C2 presented less dementias (0,5% versus 3% in C1 and 10% in C3) and delusional disorders, but more bipolar disorders (24% versus 15% in C1 and C3); C3 represented less episodes due to substance abuse (alcohol or others) and personality disorders. In both C2 and C3, no psychiatric consultation happened in the 3 months prior admission to around 40% of episodes, and 50% had stopped medication. The majority had only oral medication. Almost 24% of C2 tested positive for cannabinoids, with no differences regarding other substances.

**Conclusions:**

These findings allow the definition of 2 kinds of DTP, which present unique characteristics but some common features (namely poor adherence to consultations and are in therapeutic compliance). An assertive multidisciplinary approach, focused on current treatment and relapse prevention (including social structures, more frequent clinical follow-up, and rehabilitation centers), will be the key to their treatment.

**Disclosure of Interest:**

None Declared

